# Effect and significance of incorporating access in estimating the number of required physicians: focus on differences in population density in the target area

**DOI:** 10.1186/s12942-021-00274-0

**Published:** 2021-05-17

**Authors:** Tatsuya Suzuki, Soichi Koike, Masatoshi Matsumoto

**Affiliations:** 1 Program in Architecture, Civil and Environmental Engineering, Department of Engineering and Design, Kagawa University, 2217-20 Hayashi-cho, Takamatsu, Kagawa 761-0396 Japan; 2 Division of Health Policy and Management, Center for Community Medicine, Jichi Medical University, 3311-1 Yakushiji, Shimotsuke, Tochigi 329-0498 Japan; 3 Department of Community-Based Medical System, Faculty of Medicine, Hiroshima University, 1-2-3 Kasumi, Minami-ku, Hiroshima, 734-8553 Japan

**Keywords:** Accessibility, Capacity, Fairness, Medical resource allocation, Number of doctors, Primary care

## Abstract

**Background:**

Geographical imbalances in the health workforce, particularly the shortage of health care workers in rural areas, is an issue of social and political concern in most countries. Estimating the number of required doctors is essential for evidence-based health policy planning. In this study, we propose two methods for estimating the number of required doctors using a simple method. One is counting by unit and the other is incorporating access to medical institutions. The purpose of this study is to verify the need to incorporate access to medical institutions when estimating the number of required physicians in a region by comparing both estimation methods from the viewpoint of regional population density.

**Methods:**

We calculated the ratio of outpatients who can access medical institutions and the number of required physicians using the travel time by car and the number of patients who can be treated per doctor per day (estimation method for the number of physicians based on the access simulation, hereinafter referred to as EAS). We compared the results of this estimation with those of a conventional method, such as the number of doctors per population (estimation method for the number of physicians based on the number of patients, hereinafter referred to as ENP) to show how important it is to incorporate the element of accessibility in such a simulation analysis. Based on the results, we discussed the applicability of the proposed method.

**Results:**

ENP estimated that 38,685 outpatient primary care (PC) physicians were required and EAS estimated that 46,378 were required. There was a difference of about 8000. A comparison of the EAS-estimated number of physicians and the ENP-estimated number of physicians showed that the ENP-estimated number was small, particularly in areas with low population density.

**Conclusions:**

The results showed that it is effective to use the proposed EAS method for the estimation of PC physicians, particularly in areas with low population density. We showed that the method of allocating the number of physicians in proportion to the number of patients in a certain unit requires paying attention to the setting of the unit.

## Background

The appropriate allocation of physician resources is an urgent need in countries around the world to achieve universal health coverage [1]. The World Health Organization estimated that the shortage of health care workers in 2013 was about 17.4 million, of which almost 2.6 million were doctors [2]. Even in developed countries, there still exist urban–rural disparities [3–5]. Estimating the number of doctors needed is essential for evidence-based health policy planning. However, estimating people's demand for physicians is more difficult than estimating physician supply because many factors and assumptions are needed to perform the estimation [6–8]. Additionally, because it is difficult to understand the potential healthcare demand and the demand driven by the supply of healthcare, the demand for doctors is often discussed based on the relative difference between regions (uneven distribution of doctors).

Conventionally, the evaluation of the equity of physician distribution has been conducted using the number of physicians per unit population in an area, such as a municipality, prefecture or planned medical area [9, 10]. However, the balance between supply and demand for medical care is rapidly changing, which is caused by the overconcentration of the population and economy in cities, population outflow, and aging in suburbs and rural areas. In this scenario, primary care (PC) physicians who provide PC at clinics close to the patient's home for various diseases have been regarded as more important in recent years; that is, not only is the number of medical institutions or physicians important, but also the geographical location and accessibility of medical institutions. It is unreasonable to use only the ratio of the number of doctors to the number of patients in a region as an objective index for the appropriate placement of doctors in response to such social changes.

In previous studies, the two-step floating catchment area, gravity model and other approaches that model in detail the supply and demand of healthcare facilities that incorporate access [11, 12] were developed. These methods determine which medical institution a patient will use based on variables such as the distance to the medical institution and the capacity of the medical institution when there are multiple medical institutions in the vicinity. These detailed methods are useful for analyzing the detailed supply and demand of medical care in areas such as dense cities. However, the drawback is that it takes time and effort to target a wide area, such as an entire country. For medical policy, such as plans to allocate medical resources appropriately, it is useful to classify the entire country as the area in which analysis by such detailed methods is necessary and the area in which simple analysis is sufficient instead of detailed analysis.

Therefore, the purpose of this study is to clarify which areas can be analyzed using a simple method and which areas require the analysis of access to medical institutions when estimating the number of required doctors in an entire country. This answers the following questions when estimating the PC physicians required, considering outpatient access to medical institutions, which is an important aspect of PC: How effective is the traditional method of counting by unit? In which areas should detailed methods of measuring access be used? We propose two methods for estimating the number of required doctors using a simpler method. One is counting by unit and the other is incorporating access to medical institutions. In particular, we focus on outpatients who require PC and consider this based on differences in regional population densities.

## Methods

### Data

We obtained the address and number of physicians for each medical institution in Japan from the 2014 Survey of Physicians, Dentists and Pharmacists [13] and 2014 Survey of Medical Institutions [14]. These data are not publicly available, and were obtained with the permission of the Ministry of Health, Labour and Welfare (MHLW). We also obtained the number of outpatients and the population by age group, prefecture and area of practice from data published in the 2017 Patient Survey [15] and 2017 Dynamic Survey of Medical Institutions [16]. The population distribution was mapped using data from the National Census (Population Census 2015) [17] and the ArcGIS Geo Suite: Road Network [18] provided by ESRI Japan to measure the travel time.

The population census is conducted every 5 years, and the latest year for available data at the time of writing this paper was 2015. Therefore, we adopted other data for survey years that were close to 2015.

### Estimating the number of patients

A new board certification system for the Japanese Medical Specialty Board was introduced in 2018. ‘General practice’ was added as the 19th basic area of board certification. The discussion on how many physicians are expected to choose “general practice” is a key health policy debate at the present time, in addition to demand for specialists in other areas. The lack of specialized training for PC has been recognized as a serious problem in Japan, with internists and pediatricians serving as providers of PC. Estimating the demand for PC is difficult in the absence of a PC physician. Because of this scenario, we assume that the number of patients that require PC is equal to the number of outpatients seen by physicians with the specialty of internal medicine. There are two reasons to focus only on outpatients. The first is that, in Japan, where there are no PC physicians, patients may go to the internal medicine outpatient department for PC. The other reason is that the interpretation of the analysis results is simply the number of outpatients per day, unlike inpatients, which requires a doctor for an arbitrary period of time.

First, we extracted the daily number of outpatients per population. The population was extracted by age, gender and prefecture of residence, and we extracted hospitals and clinics separately from the 2017 Patient Survey. The average value of these data by hospital and clinic is shown in Table 1.

**Table 1:** Daily number of outpatients per population

	OP for hospital/100 k pop		OP for clinic/100 k pop	
	Average		Average	
Age	Male	Female	Male	Female
0–4	1132.72	998.02	5445.72	5257.64
5–14	549.19	399.81	2375.87	2179.17
15–24	344.36	453.36	999.47	1477.72
25–34	430.13	872.70	1074.62	2447.02
35–44	596.23	902.85	1320.47	2312.89
45–54	907.57	1102.21	1758.53	2538.28
55–64	1497.47	1468.66	2627.15	3375.38
65–74	2436.87	2147.47	4330.66	5223.89
75 and over	3468.00	2768.57	6720.62	7113.83

Next, we extracted the ratio of internal medicine patients to total patients from the 2017 Dynamic Survey of Medical Institutions. The national average value of these data is shown in Table 2.

**Table 2:** Ratio of internal medicine patients to total patients

	Internal medicine/all clinical departments	
	Average	
	Hospital	Clinic
Outpatients	0.22	0.30

Finally, we extracted the population distribution by age and sex aggregated for each approximate 1 km mesh from the Population Census 2015. The 1 km mesh is a grid divided by a latitude of 30 s and a longitude of 45 s. It is called the 1 km mesh because it covers an area of about 1 km square. This is a statistical unit that is standardized in Japan, and the most commonly used aggregation unit in national statistics. Although data aggregated by administrative division are also disclosed in the census, they differ in size between urban and suburban areas, and are too wide for measuring access to regional facilities, particularly in rural areas. Therefore, in this study, because it is necessary to understand the distribution of patients using a fine aggregation unit when measuring access to medical institutions, the 1 km mesh was selected as the unit for counting the number of patients.

Using the above data, the estimated internal medicine outpatients for each 1 km mesh is calculated using the following equations:

1 $${hP}_{i}={\sum }_{p}\sum_{age}\sum_{s}{POP}_{i, s, age}{hR}_{p, s, age}{x}_{ip},$$

2 $${cP}_{i}={\sum }_{p}\sum_{age}\sum_{s}{POP}_{i, s, age}{hR}_{p, s, age}{x}_{ip},$$

$hP$: number of hospital outpatients, $cP$: number of clinic outpatients, *hR*: daily number of hospital outpatients per population, *cR*: daily number of outpatients for clinic per population, *i*: any 1-km mesh, $\forall i\in I, n(I)=387061$, *p*: any prefecture, $\forall p\in P, n(P)=47$, *age*: any age group, $\forall age\in AGE=\left\{0-4, 5-14, 15-24, 25-34, 35-44, 45-54, 55-64, 65-74, 75-\right\}, n\left(AGE\right)=9$ *s*: Any sex, $\forall s\in S=\left\{male, female\right\}, n\left(S\right)=2$, ${x}_{ip}$: binary variable that indicates whether mesh *i* is included in prefecture *p,* ${x}_{ip}\in \left\{0, 1\right\}$

3 $${K}_{i}={hK}_{i}+{cK}_{i},$$

4 $$h{K}_{i}={{\sum }_{p}{hIM}_{p} {hP}_{i}x}_{ip},$$

4 $$c{K}_{i}={{\sum }_{p}{cIM}_{p} {cP}_{i}x}_{ip},$$

*K*: daily number of internal medicine outpatients, *hK*: daily number of hospital internal medicine outpatients, *cK*: daily number of clinic internal medicine outpatients, *hIM*: ratio of hospital internal medicine outpatients to total hospital outpatients, *cIM*: ratio of clinic internal medicine outpatients to total clinic outpatients.

### Setting the capacity of physicians

Article 19 of the Ordinance for Enforcement of the Medical Care Act stipulates that, in a general hospital, there should be one doctor for every 40 outpatients per day. At the present time, doctors are assigned according to this rule. We set $Pcap$ to 40 based on the above. We calculated the number of outpatients that can be treated in a day at each medical institution (${CAP}_{j}$) using the following equation:

6 $${CAP}_{j}=Pcap {D}_{j},$$

*j*: any medical institution with a physician, $\forall j\in J, n(J)=51523$, $Pcap$: maximum number of outpatients that a physician can treat in a day (= 40), ${D}_{j}$: number of physicians in medical institution *j.*

Additionally, the capacity of each medical institution may be affected by equipment. Therefore, we assume that the number of physicians in each medical institution does not exceed the current number of physicians.

### Methods for estimating the number of required physicians

**Fig. 1:**
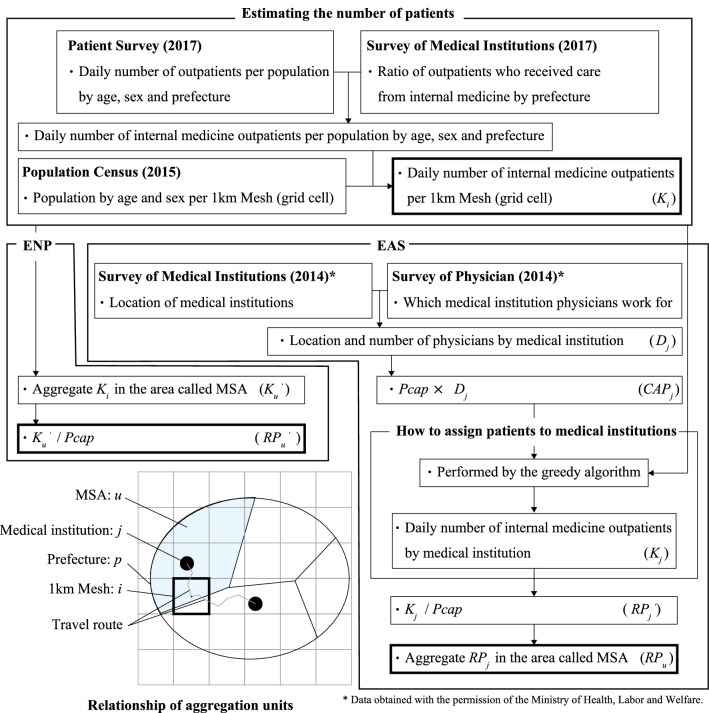
Flow chart of methods for estimating the required number of physicians

In this study, we compare EAS, which is a method for estimating the number of required physicians incorporating access to medical institutions, and ENP, which estimates the number of required physicians in proportion to the number of patients. The specific details of each estimation method are presented below. Additionally, Fig. 1 shows a flow chart of the methods.

### How to assign patients to medical institutions for EAS

We calculated the travel time on the assumption that all outpatients in each 1 km mesh, which is a unit surrounded by about 1 km on all sides, traveled on the road route to the medical institution starting from the center point of the mesh. We assumed that the travel route is the shortest route using Network Analyst, which is a tool of ArcMap (Ver. 10.7, ESRI Japan, Tokyo, Japan, 2018), and set the travel speed of cars using the speed limit of each road.

The assumption is that outpatients that require PC choose a medical institution close to their place of residence. The location-allocation problem with the minimization of the total travel time shown by the following Eq. (7) as the objective function is solved.

There are many definitions of accessibility to medical resources [19]. In Japan, everyone has insurance, and there are no restrictions on the medical institutions that patients can attend; that is, there are no financial restrictions or area restrictions, and patients are free to choose a medical institution. For this reason, accessibility in this study is defined only by travel time, which is one of the most fundamental definitions. The calculation is expressed by the following equation:

7 $$minimize\; T=\sum_{i}\sum_{j}{K}_{i} {x}_{ij}{t}_{ij},$$

8 $$\sum_{i}{K}_{i} {x}_{ij}{y}_{ij}\le {CAP}_{j}\left(0\le {x}_{ij}\le 1\right),$$

9 $${y}_{ij}= \left\{\begin{array}{ll}0&{t}_{ij}>{t}_{max}\\ 1& { t}_{ij}\le {t}_{max}\end{array}\right.,$$

10 $$\sum_{j}{x}_{ij}\le 1,$$

$T$: total travel time to the medical institution, ${x}_{ij}$: percentage of patients living in mesh *i* who use medical institution *j,* ${t}_{ij}$: travel time from mesh *i* to medical institution *j,* ${t}_{max}$: upper limit of the travel time (= 30), ${y}_{ij}$: binary variable that indicates whether travel time constraints have been satisfied.

We performed the calculation using the greedy algorithm, which is an approximation algorithm. We prioritized patients with short travel times and assigned them to the nearest medical institution. Equation (8) indicates that the total number of outpatients using medical institution *j* does not exceed ${CAP}_{j}$. Additionally, ${x}_{ij}$ is the ratio of outpatients in mesh *i* who can receive medical treatment at medical institution *j* to all outpatients in mesh *i*. ${y}_{ij}$ is a binary variable based on the travel time by car to the medical institution. As shown in Eq. (9), we set the maximum travel time to the available medical institution, and if outpatients can reach the medical institution within that time, ${y}_{ij}$ is 1; otherwise, ${y}_{ij}$ is 0. We set ${t}_{max}$ to 30 min. with reference to the daily living area defined as an integrated community care system that seamlessly provides healthcare, long-term care, prevention, housing and livelihood support services so that senior citizens can live independently in their communities [20]. If a patient cannot reach one outpatient medical institution, the patient is considered for treatment at another outpatient medical institution if it is within the time threshold. Equation (10) shows the ratio of outpatients who can receive medical treatment at any medical institution to all outpatients in mesh *i*. If it is less than 1, this indicates that some outpatients do not have an available medical institution.

We performed these calculations using Java Eclipse MARS4.5.

### Estimating and aggregating the number of physicians

We calculated the minimum number of required PC physicians by dividing the number of patients calculated in the previous section by the number of patients that physicians can treat in a day. Therefore, we expressed the number of required outpatient PC physicians for medical institution *j* (${RPout}_{j}$) using the following equation:

11 $${RP}_{j}=\lceil\frac{\sum_{i}{K}_{i} {x}_{ij}{y}_{ij}}{Pcap}\rceil,$$

In this study, the estimated number of outpatient physicians was aggregated in an area. In Japan, there are three administrative units: municipalities, prefectures and country, and there are approximately 1700 municipalities and 47 prefectures. There are three planning areas in medical care: the primary medical area based on the municipality, the secondary medical area that consists of several municipalities and the tertiary medical area based on the prefecture. We used the secondary medical area [hereinafter referred to as the medical service area (MSA)], which is a medical administration area created by planning complete inpatient care. The number of required physicians RP using EAS in any MSA is as follows:

12 $${RP}_{u}={{\sum }_{j}{RP}_{j}x}_{ju},$$

13 $$\sum_{u}{x}_{ju}= 1,$$

*u*: any MSA, $\forall u\in U, n(U)=344$, ${x}_{ju}$: binary variable that indicates whether medical institution *j* is included in MSA *u,* ${x}_{ju}\in \left\{0, 1\right\}$.

By contrast, ENP estimates the number of physicians in proportion to the number of patients. The number of patients in any MSA is calculated by aggregating the number of patients in any 1 km mesh, as shown in Eq. (14), by MSA. Additionally, there are 344 MSAs, and their areas vary between urban areas and rural areas. However, even the smallest MSA contains 36 meshes, and the largest MSA contains 11,371 meshes, so the 1 km mesh is a unit of aggregation that is sufficiently smaller than the MSA. Some meshes straddle two or more MSAs at the boundary of the MSA. In this study, because the patient distribution is the center point of the 1 km mesh, we aggregate it in the MSA that includes the center point. The calculation is expressed by the following equation:

14 $${K}_{u}^{^{\prime}}={{\sum }_{i}{K}_{i}x}_{iu},$$

${x}_{iu}$: binary variable that indicates whether mesh *i* is included in MSA *u,* ${x}_{iu}\in \left\{0, 1\right\}$.

The number of required physicians estimated by ENP is the number obtained by dividing ${K}_{u}{^{\prime}}$ by $Pcap$ and rounding up to the nearest whole number:

15 $${RP}_{u}^{^{\prime}}= \left\lceil\frac{{Kout}_{u}{^{\prime}}}{Pcap} \right \rceil.$$

### Analysis

We aggregated the number of required physicians calculated by the EAS method shown in Eq. (12) and ENP method shown in Eq. (15) for each MSA (all 344 areas). We also aggregated the actual number of PC physicians by MSA for comparison. Furthermore, to confirm the relationship between regional characteristics that result from population density and the difference between estimates, we classified MSAs into three categories based on the population density (first tertile 136.56 pop./km^2^, second tertile 489.74 pop./km^2^). We used scatter plots to show the relationship between the estimated number of required physicians and the population density. We also showed the difference between estimates using EAS-estimation and ENP-estimation.

Next, we compared the fairness of the distribution of required PC physicians estimated by EAS and ENP using the Gini coefficient. The Gini coefficient based on the cumulative patient ratio and cumulative physician ratio represents “quantity fairness” because it assumes that a consistent ratio of physicians working in an area to patients living in an area is fair. ENP is an estimation method that calculates the number of required PC physicians as a ratio of the number of PC physicians to the number of patients for each MSA; hence, it can be regarded as an estimation method that matches “quantity fairness.” By comparing the Gini coefficients of EAS and ENP, we observed how the EAS estimation reduced “quantity fairness.”

Furthermore, we investigated the effects of changing the upper limit of travel time ${t}_{max}$ and the maximum number of outpatients $Pcap$ that a physician can treat in a day, which are important factors in estimating the number of required PC physicians using EAS.

## Results

### Quantitative comparison

The higher the population density, the more actual PC physicians there were for the number of patients. We found that the lower the population density of areas, the lower the ratio of the number of actual physicians to the number of patients.

Additionally, Table 3 shows the difference between the number of required PC physicians estimated by EAS and ENP. There were 38,685 physicians required using ENP and 46,378 physicians using EAS, which is about 44% and 52% of the actual number of physicians. The difference between the number of required PC physicians estimated by EAS and ENP is about 8000.

**Table 3:** Number of patients and doctors in MSAs for the three categories

Type of MSA	Number of MSAs (type of MSA/total)	Total population (type of MSA/total)	Number of		Estimation by access simulation: EAS	Estimation by number of patients: ENP
					Number of physicians for outpatients (type of MSA/total)	Number of physicians for outpatients (type of MSA/total)
			Outpatients (type of MSA/total)	Actual physician (type of MSA/total)		
High population density	114 (33.1%)	85,675,469 (66.8%)	1,004,552 (65.2%)	58,982 (66.7%)	28,959 (62.4%)	25,178 (65.1%)
Middle population density	115 (33.4%)	29,466,441 (23.0%)	369,793 (24.0%)	20,824 (23.6%)	11,936 (25.7%)	9305 (24.1%)
Low population density	115 (33.4%)	13,218,347 (10.3%)	165,658 (10.8%)	8609 (9.7%)	5483 (11.8%)	4202 (10.9%)
Total	344 (100.0%)	128,360,257 (100.0%)	1,540,003 (100.0%)	88,415 (100.0%)	46,378 (100.0%)	38,685 (100.0%)

### Comparison of methods for estimating the number of physicians

**Fig. 2:**
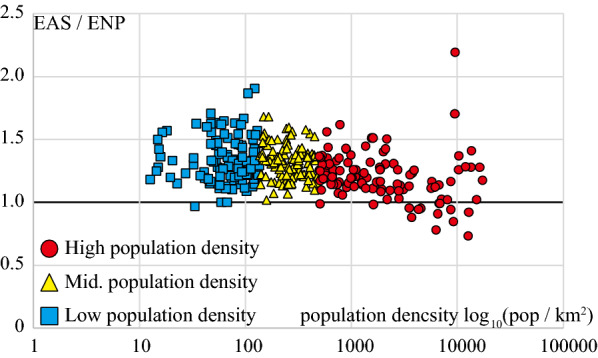
Relationship between the population density and difference in the required number of PC physicians estimated by EAS and ENP. The vertical axis of the scatter plot shows the ratio of the required number of PC physicians estimated by EAS to the required number of PC physicians estimated by ENP. The horizontal axis shows the population density of each MSA on a logarithmic scale

A comparison of the number of required PC physicians using the two estimation methods demonstrated that the number of required PC physicians estimated by EAS was generally 1–1.5 times that estimated by ENP (Fig. 2).

**Fig. 3:**
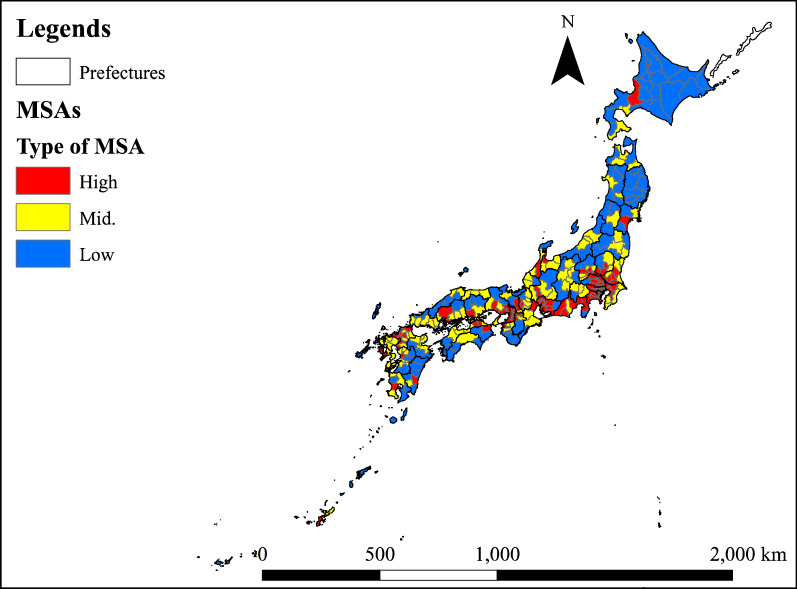
Types of MSAs

**Fig. 4:**
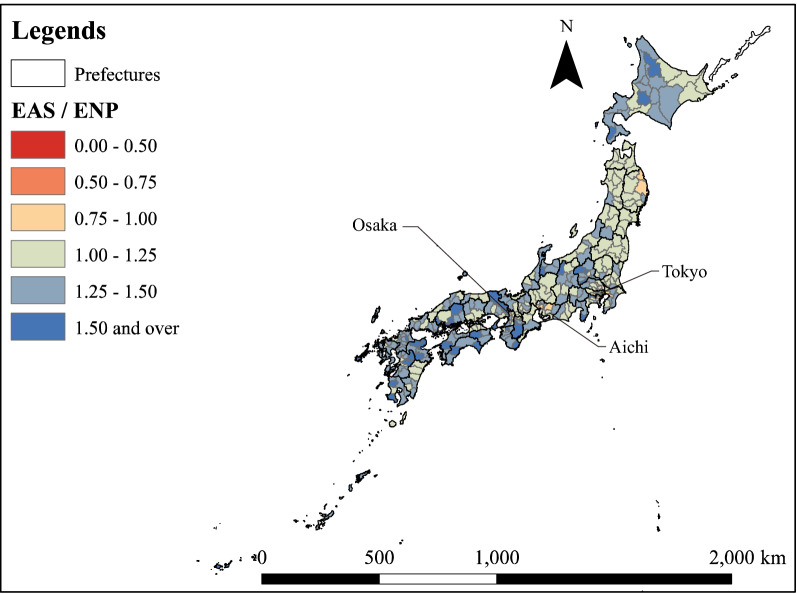
Distribution of the ratio of the required number of PC physicians estimated by EAS to that estimated by ENP

Figure 3 shows the spatial distribution of MSAs divided into three categories according to population density. Figure 4 shows the spatial distribution of the ratio of the number of required PC physicians estimated by EAS to the number of required physicians estimated by ENP. MSAs with an EAS/ENP of less than 1 are distributed around the three major cities of Tokyo, Aichi and Osaka. Additionally, there are more MSAs with high EAS/ENP values on the west side than on the east side.

**Fig. 5:**
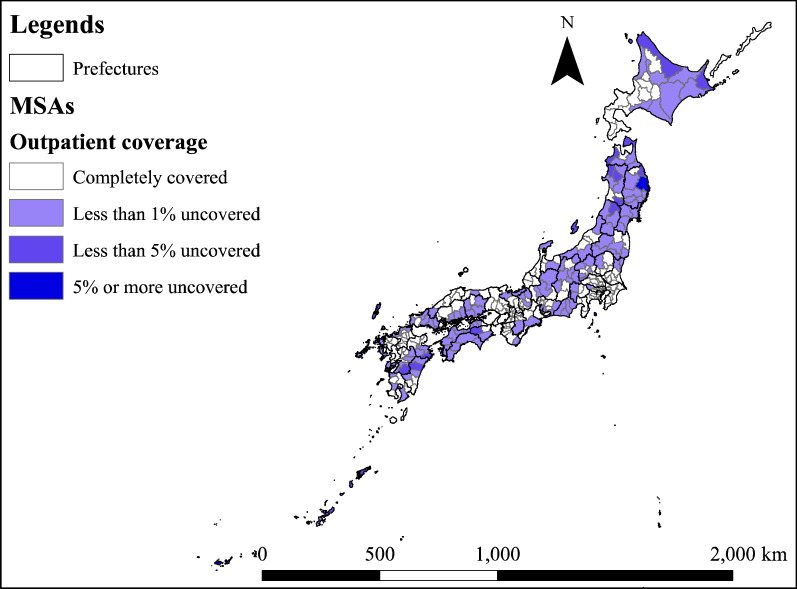
Distribution of outpatient coverage estimated by EAS

Figure 5 shows a map of the percentage of patients who cannot reach the medical institution by the upper limit of the travel time (= 30 min.) in the process of estimating the number of required PC physicians using EAS. The MSAs in which about 1% of patients cannot use medical institutions except in large cities are spread widely. In some MSAs, more than 5% of patients do not have access to medical institutions.

The Gini coefficients of the number of required PC physicians estimated by ENP and the number of required PC physicians estimated by EAS are 0.00271 and 0.07946, respectively. Because ENP is a method that estimates the number of required PC physicians in proportion to the number of patients, the Gini coefficient of ENP is close to 0.

By changing the upper limit of the travel time (${T}_{max}$) and the maximum number of outpatients $Pcap$ that a physician can treat in a day, we obtained the following three findings (Table 4).

**Table 4:** Ratio of the number of required PC physicians estimated by EAS to ENP for different conditions (EAS/ENP)

	*Pcap* = 30	*Pcap* = 40	*Pcap* = 50
*T*_*max*_ = 20 min			
High population density	1.043	1.083	1.116
Mid. population density	1.074	1.144	1.202
Low population density	1.073	1.147	1.205
*T*_*max*_ = 30 min			
High	1.043	1.083	1.116
Mid.	1.076	1.145	1.202
Low	1.080	1.150	1.207
*T*_*max*_ = 40 min			
High	1.043	1.083	1.116
Mid.	1.077	1.146	1.202
Low	1.082	1.152	1.207

*T*_*max*_ Upper limit of the travel time, *Pcap* Maximum number of outpatients that a physician can treat in a day

The lower the population density in an area, the smaller the number of required PC physicians estimated by ENP than EAS. The longer the access time set in the method, the smaller the number of required PC physicians estimated by ENP than EAS. If the PC physician’s medical capacity was set to a low value, the difference between ENP and EAS was small.

**Fig. 6:**
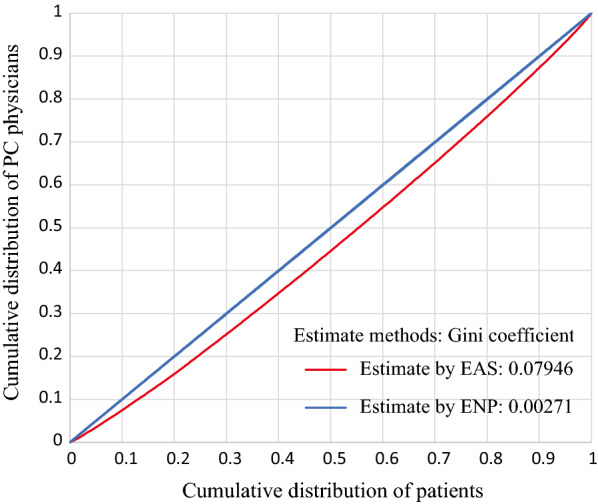
Ratio of the number of required PC physicians estimated by ENP to that estimated by EAS (${T}_{max}=40, Pcap$= 50)

Figure 6 shows the spatial distribution of the ratio of EAS to ENP when ${T}_{max}=40$ and $Pcap=50$ are set. Only a few of the big cities in Tokyo and Osaka have a number of required PC physicians estimated by ENP that is less than the number of required PC physicians estimated by EAS.

## Discussion

### Comparison of the number of required PC physicians estimated by EAS and that estimated by ENP

A comparison of the number of required PC physicians estimated by EAS and that estimated by ENP in the three categories showed that the former was larger than the latter in almost all regions. This tendency was more pronounced in areas with low population densities. However, according to the required number of doctors estimated by EAS, there were some uncovered areas where there were patients who could not reach a medical facility within 30 min. by car. In the uncovered area, there are about 1300 outpatients, which is about 0.1% of the total outpatients, and more PC physicians are needed. In many previous studies, it was shown that physicians tend to be in short supply in these low-density areas and underserved areas [21]. By contrast, it has been indicated that non-physician clinicians, not doctors, make a large contribution to preventive care services for the elderly [22]. Therefore, there are several possible solutions to the uneven distribution of medical resources, but it can be said that the EAS method in this study is also effective in that such areas can be identified.

### Implications of the difference between the two estimation methods

**Fig. 7:**
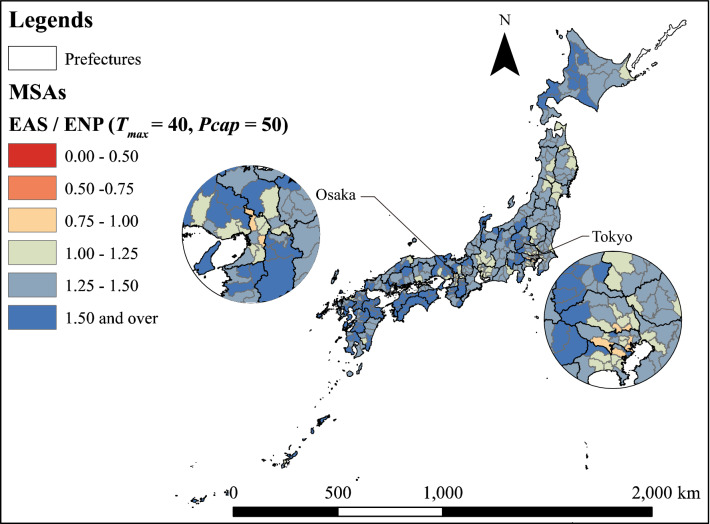
Gini coefficients calculated using the cumulative number of patients and cumulative number of physicians

The difference between the two estimation methods depends on whether outpatients’ access to medical institutions is included; that is, it shows the effect of ignoring access when allocating PC physicians based on “quantity fairness.” The Lorenz curve shown in Fig. 7 is represented by a straight line in which the ideal “quantity fairness” is evenly distributed between the number of physicians and the number of patients. The results show that the estimation by ENP achieves the minimum Gini coefficient obtained when it is calculated in the aggregation by MSA. By comparison, the estimation that incorporates factors other than the quantity of access moves away from “quantity fairness,” but EAS in this study can obtain a Gini coefficient of about 0.08.

In this regard, Talen and Anselin [23] highlighted the problem of evaluating placements using counts by unit. If access is not complete within the region, that is, if the destination is not in the area, fairness indicated by counting by unit, such as the Gini coefficient, should be interpreted with caution.

The above suggests that such a Gini coefficient is effective when registering a medical institution that can provide medical care, such as the general practitioner system represented by the United Kingdom. However, if patients can freely select medical institutions, such as in Japan, which is the subject of this study, it is necessary to verify whether the service area and aggregation unit match. Talen and Anselin also indicated that the problem can be avoided if the appropriate units are used when assessing fairness using counts by unit [23]. In this study, we compared the number of required PC physicians estimated by ENP with the MSA as the aggregation unit and the number of required PC physicians estimated by EAS with the service area set by the travel time. The results of this study suggested that the MSA was too large for estimating the number of outpatient PC physicians using the count-by-unit method. This indicates that we should carefully interpret the results of studies ([24, 25] etc.) that attempt to evaluate the uneven distribution of doctors using the Gini coefficient.

### Toward an expansion to various clinical departments and regions

Variables ${cout}_{\mathrm{max}}$ and ${T}_{max}$ should be set to different values depending on the department in the field of medical care, and should be set to be feasible depending on the region and scenario. For example, Table 4 with varying settings for ${cout}_{\mathrm{max}}$ and ${T}_{max}$ shows that when medical examinations are efficient as a result of using information and communications technology, the supply capacity improves, so ${cout}_{\mathrm{max}}$ increases. The larger ${cout}_{\mathrm{max}}$, the larger the difference between the number of required PC physicians estimated by EAS and ENP. If the travel time is replaced with the patient’s permissible travel burden (e.g., cost, time, labor), ${T}_{max}$ increases because of the spread of self-driving cars and the reduction of the access burden, such as the introduction of patient transporters. The larger ${T}_{max}$, the larger the difference between the number of required PC physicians estimated by EAS and ENP; that is, when there are social conditions or regional characteristics for which ${cout}_{\mathrm{max}}$ and ${T}_{max}$ become large, it becomes more meaningful to incorporate access into the estimation of the number of required PC physicians.

Additionally, we consider that the estimation method for the number of required PC physicians based on the access simulation will greatly contribute to the evaluation of the placement of PC physicians and the estimation of the required number where it is important to ensure outpatient access. Our method can identify the medically served area and underserved area by calculating solutions for the entire country using the same standard. Particularly in the underserved area, the possible solutions differ depending on whether the number of doctors is insufficient or the location of the medical institution is the reason that the area is underserved. When considering such regional medical measures, it is useful to first understand the whole country, and a simple method is necessary from a practical point of view. The results of this analysis will be helpful for understanding areas that have problems with the balance of medical supply and demand in many countries with problems of uneven medical resource distribution caused by population concentration in urban areas and population decline in rural areas [26–28].

### Limitations

This study has three limitations. First, because the estimation of the number of required PC physicians is based on the current location of medical institutions, it is not possible to determine the efficiency improvement achieved by a particular number of required PC physicians using the optimal location of medical institutions.

Second, because the estimation results change depending on the size of the unit to be aggregated and the uniformity of the population, the number of required PC physicians obtained in this study and the difference between EAS and ENP cannot be generalized. The same can be said for the number of patients and access estimated using the 1 km mesh. However, although more detailed access can be measured using a finer mesh, we believe that the effect of outliers will be greater in terms of estimating the number of patients. The verification of the appropriate resolution of the mesh is a topic for future research.

Additionally, the definition of access to care requires more variables that depend on the health care system in each country or area. The travel time of cars along the road network considered in this research is just one factor. To apply this research method to other areas, it is important to add elements according to social conditions, such as the public transportation network, cost and selection of medical institutions according to disease severity.

## Conclusions

The proposed method for estimating the number of required PC physicians called EAS is suitable for estimating and evaluating the number of required PC physicians that specialize in outpatient departments. This estimation method evaluates both efficiency from the viewpoint of the number of required PC physicians per patient and fairness in terms of ensuring uniform access. In the context of uneven medical resource distribution, the number of doctors per population is often used as an indicator of appropriate allocation. In this study, we clarified the size of the difference between the number of required PC physicians based on simulations incorporating access and without access, and in what areas it is likely to appear.

In countries with an uneven medical resource distribution and dispersed population distribution, it is not possible to correctly establish a medical care provision system based only on “quantity fairness.” The results of this study provide suggestions on selecting methods when estimating the number of physicians needed and when assessing imbalances in medical resources in different countries and regions.

## Data Availability

The data that support the findings of this study are available from the MHLW repository https://www.mhlw.go.jp/toukei/list/33-20.html and https://www.mhlw.go.jp/toukei/list/79-1.html but restrictions apply to the availability of these data, which were used under license for the current study, and so are not publicly available. Data are however available from the authors upon reasonable request and with permission of the MHLW. The datasets generated and/or analyzed during the current study are available in the ESRI JAPAN repository, https://www.gisdata-store.biz/product/1632/.

## Abbreviations

EAS: Estimation method for the number of required PC physicians based on the access simulation
ENP: Estimation method for the number of required PC physicians using only the number of patients
MHLW: Ministry of Health, Labour and Welfare
MSA: Medical service area
PC: Primary care
